# A Qualitative Inquiry into Nursing Students' Experience of Facilitating Reflection in Clinical Setting

**DOI:** 10.1155/2017/6293878

**Published:** 2017-04-04

**Authors:** Shahnaz Karimi, Fariba Haghani, Nikoo Yamani, Majid Najafi Kalyani

**Affiliations:** ^1^Department of Medical Education, Medical Education Research Center, Isfahan University of Medical Sciences, Isfahan, Iran; ^2^School of Nursing, Fasa University of Medical Sciences, Fasa 74616 86688, Iran

## Abstract

*Background and Aim*. Reflection is known as a skill that is central to nursing students' professional development. Due to the importance and the role of reflection in clinical areas of nursing, it is important to know how to achieve it. However, nursing trainers face the challenge of how to help their students to improve reflection in clinical settings. The aim of this study was to investigate the nursing students' experiences of facilitating reflection during clinical practice. This qualitative study was conducted by qualitative content analysis approach. Twenty nursing students during the second to eighth semester of their educational program were selected for participation using purposive sampling. Data were collected through in-depth semistructured interviews. The interview was transcribed verbatim, and qualitative content analysis was used to analyze the data. From the data analysis, four main themes were extracted. Motivation to reflect, complex experiences, efficient trainer, and effective relations were four main themes obtained from study that, in interaction with each other, had facilitating roles in students' reflective process on experiences. The findings revealed that the nursing students' reflection in clinical settings is effective in personal and professional level. Reflection of nursing students depends on motivational and educational factors and these factors increase the quality of care in patients. Furthermore, nursing educators need to create nurturing climate as well as supporting reflective behaviors of nursing students.

## 1. Introduction

Despite different models and theories proposed about reflection, there is no consensus that reflection is actually occurring or that it is leading improved practice [1]. However, reflection is more than being thoughtful and refers to a process that could help individuals to learn from their experiences [2]. This approach includes critical reviewing of experience so that it leads to change in practice and to develop personal and professional competencies in a positive way in the future [3].

During the recent years, reflection has found a more obvious role in training centers, so that it is known as a factor to gain practiced competence [4]. Based on the fact that nursing is a practice-based discipline and clinical education has an essential role in developing clinical ability of individuals, reflection is considered as a significant and valuable tool to develop professional nursing, and it is widely used in learning and teaching method in both classroom and clinical environments [5]. Reflection plays a prominent role in enabling individuals to create a real and significant relationship between education and practice [6, 7]. Nevertheless, James and Clarke (1994) stated that necessary skills for reflection are unclear and teaching reflection is hard [8]. Students also consider the reflection process laborious and believe that this process does not occur naturally, and it requires a safe and confident environment in which they can develop reflection with personnel's support [9]. Therefore, facilitating reflection and reflective abilities is considered as important components of professional development [10].

Over the past 50 years, higher-education policy makers have shown an increasing interest in reflection-based educational programs as a driving force for learning. Andersen (2016) stated that skills and capacity of students improve when they could reflect on their daily events, and it is achieved by involving students in an activity which uses their conscious experience and helps them express their thoughts [11]. It is essential to facilitate reflection by medical trainers; using written or web-based portfolios, assess the reflective work of students and identify educational barriers in medical curriculum [12]. It is particularly important to create a safe environment which reflection on experience takes place [13].

There is a wide variety of different approaches and constraints of an academic course to implement reflection in medical education. Many teaching programs insist on individuals keeping a reflective diary and often the entries are used for assessment that exposes the vulnerability of the individual [14]. It has to be expected that students out of the predetermined programs reflect on experiences too. There are no easy answers to this issue and it requires careful consideration. What are factors that could be required to facilitate and support reflection on clinical experiences? Therefore, it is necessary to identify the facilitating reflection factors on clinical experiences according to real experience as it happened by nursing students. Additionally, an understanding of nursing students' experiences on facilitating reflection is important because it will help organizations to identify the factors that improve the quality of clinical teaching. Many researchers mentioned the lack of empirical evidence to facilitate the use of reflection in nursing education and practice [15]. Thus, the specific objective of this study was to identify facilitating reflection factors based on nursing students' experience in clinical setting.

## 2. Materials and Methods

### 2.1. Research Design

This paper, which is a qualitative inquiry, is part of the larger grounded theory study entitled “Exploring the Process of Reflection in Nursing Students in Clinical Learning Environment” [16]. Content analysis approach was used in this present study so that it allows participants to describe experiences about facilitating reflection in clinical setting. Content analysis emphasizes allowing categories to emerge out of data and on recognizing the significance for understanding the meaning from the context in which an item is analyzed and the categories derived from it appeared [17].

### 2.2. Data Collection

Participants in purposeful sampling consisting of 20 students were selected from among undergraduate nursing students of medical science universities of Shiraz, Fasa, Iran, students who tended to express their experience. We selected students from the second semester, because nursing students are required to pass 36 credits in the clinical field, which start from the second semester, and they are frequently faced with various complex experiences from the beginning training program that may lead to reflection. It was attempted to select participants with the maximum variation (regarding semester, gender, average level, and marital status) in order to achieve deep and extensive data. The inclusion criteria for participants in the study were as follows: students who were enrolled in the undergraduate nursing program in Shiraz or Fasa universities, having at least one-term training in clinical learning environment, and willingness to participate and share experiences. The 1st-semester students were excluded since they do not have clinical experience. Data were gathered using in-depth semistructured interviews. Following written consent, one-to-one interviews were conducted in the Persian language, in a convenient place, and in a suitable time depending on the willingness of the participants. The length of each interview varied from 45 to 90 minutes. At the beginning of the interviews, some general questions were asked in order to get more acquainted with the participant and create more intimate situation and build more trust; then more specific questions were asked to the aim of the research. Questions were as follows: please describe one of your clinical experiences that has caused you to think and reflect more; which situation caused you more to reflect? What leads you to reflect on these situations? Then, according to the responses and in order to clarify and probe for future detail, exploratory and deeper questions were asked as follows: could you explain it more? Can you give an example? Finally, more open questions were asked as follows: do you want to add anything more? This study was conducted during 2015-2016 and continued until data saturation was reached and when all categories and subcategories were formed, no new codes or categories were added.

### 2.3. Data Analysis

Immediately after each interview, the contents were handwritten and typed. Each transcript was checked for accuracy against tapes. For immersion in data, the researcher read the transcripts and listened to each of the tapes several times to reach an overall understanding. For analysis, the data used qualitative content analysis with a conventional approach. The parts related to the experiences among the participants regarding the reflection-facilitators were extracted from the interviews. Therefore, the primary codes were extracted. Then the codes were merged and categorized according to their similarity. The categories which were similar were classified in more general categories, and each category was given a name. Finally, in the case of having a high degree of abstraction, the themes can be determined.

The rigor and trustworthiness of the qualitative data were examined using the criteria of Lincoln and Guba [18]. Credibility of finding has been established through the good interaction with the participants and long-term involvement of the researcher in the data. Thus, the researchers were involved in the study for more than one year. Member check was used for comparison of congruency between ideas derived from data and opinions of the participants. Conformability was assessed by external checker familiar with qualitative research. In external check, the extracted codes and categories were presented to the colleagues, and their appropriateness was controlled and verified and consensus was achieved in this regard. Moreover, sampling with the maximum variation contributed to data transferability and authenticity. For dependability, the researcher recorded and reported the whole research process to allow for follow-up research by others.

### 2.4. Ethical Considerations

After approval of the study by the ethical committee of Isfahan University of Medical Sciences and obtaining permission to proceed from the officials of the nursing schools of both Shiraz and Fasa Universities of Medical Sciences, the first author began to collect the data. In order to comply with ethical considerations, all interviews were conducted with prior appointment with the participants. The researcher considered all of the principles of research ethics, such as informed consent, keeping anonymity, confidentiality, and freedom to leave the study.

## 3. Results

### 3.1. Participants' Characteristics

The participants of the present study are composed of twenty undergraduate nursing students in 2nd to 8th semesters with average age of 22.3 among whom twelve female and eight male students attended. Passing at least one training course in the clinical setting and tendency to attend the survey were the requirements of this experiment.

### 3.2. Analysis of Interviews with Students

Four main themes and a number of subcategories were identified in the data. These are represented in Table 1. Each will be described with the help of illustrative quotations from participants.

### 3.3. Motivation to Reflect

Most of the participants said that they reflected on clinical experiences from a defined point that is lack of knowledge or feeling a need that did not know about it at the time. The reflection on experience originated from different motivations in either case. This theme can be broken down into subcategories: desire to know more, sense of accountability, self-actualizing tendency, and personal values.

#### 3.3.1. Desire to Know More

Students' desire to gain more knowledge in clinical practice was one of the effective factors in the reflection process on clinical experience. Students in encountering unknown conditions began to reflect on clinical experiences and gather information from different sources to eliminate the knowledge needs. Many participants desired to provide an effective care by developing their own knowledge. Finding out about desire to know more for reflection on experience was clearly high for participants.

In this regard, one of the participants (p7) stated the following: “a patient referred to the clinic and was diagnosed to have pulmonary embolism. I should learn to frame and reframe nursing care for this complex diagnosis, and then modify our actions. I felt a little something. This desire led me to come back on my experience.”

Another participant (p11) said that “my main problem in the clinical practice is the lack of practical knowledge to meet the needs of a unique patient. I need to provide better nursing care through assess of my practical experience.”

In the first of those participants' quotations, reflection on experience anticipated that she will not surely be able to care for a patient and desire to know more knowledge. The second participant had reflected on clinical experience. Both of participants felt they could not care of patient and desire to gain more essential knowledge.

#### 3.3.2. Sense of Accountability

Most participants reacted with a patient's need to accountability. They said that the need to respond to the patient has provided a dynamic context for reflection on experience. Some expressed distress of nonaccountability to the patient, which may have delayed nursing care for patient and caused damage to them.

In this regard, one of the participants (P6) stated that “I really felt unwilling of myself, that I as a nurse with at least three years of academic study cannot respond to my patient's questions, so what should I expect from myself? I thought that I could try at least not to be ashamed of myself anymore.”

Another interviewee (p15) said that “sometimes the questions of the patients have led me to continue my training course. Those questions encouraged me to seek more knowledge. Because the need for responding to patients directs us toward our weaknesses, then we come back again and seek to overcome these weaknesses.”

The participants talked about their lack of knowledge for accountability to patient and patient's family. Participants mentioned that they cannot assess the patients' needs; therefore they cannot reflect on their experiences.

#### 3.3.3. Self-Actualizing Tendency

All the students emphasized the central role of their own will in reflection in comparison to other factors. Many participants desired to reflect on clinical experiences due to what is in their minds or hearts. In relation to the important role as the self-actualizing tendency in reflection on clinical experiences, the 13th participant stated that

trainer is a step for me; clinical teaching is a step for me; all the available facilities are steps for me; all of these are underlying situations for me, but I cannot go up these stairs, unless I want and intend to self-improvement.

Another participant (P6) said that

at the very beginning for reflection on experience, self-tendency is important. It is said that you can waken up, he who is asleep, but you cannot waken up, he who pretends to be asleep. Nothing is possible, unless we want.

In relation to the importance of student's tendency in reflection, another participant (P11) stated that “I know about my weakness, I want to grow and attempt to learn from the experiences and improve myself.”

#### 3.3.4. Personal Values

Almost all the students participating in this qualitative study believed that their values were considered as a first priority for reflection. The issue of religious beliefs was important for the participants. They stated some beliefs such as “we'll see the outcome of our behavior in this world” and “I should like for my patients whatever I like for myself, because there may come some days that my family or I might be sick.” These beliefs increase responsibility in reflection on clinical experiences. In this regard, one of the participants (P17) stated that

I believe that whatever I do for my patients will have a feedback on my own life, and I may experience it and God will compensate it for me in another situation. Therefore, I think about whatever I do for my patients to make sure that I have done my best for my patients.

Another participant (P14) stated that

my patient has some rights, and I'm not allowed to let her feel more pain or let his treatment last longer. This encourages me to learn from my experience and have better performance in other situations.

### 3.4. Complex Experiences

According to their experience, participants emphasized that some factors related to the patients such as including disease characteristics and some characteristics of the patient have essential roles in facilitating the reflection process. They stated that they had reflected more in case of rare or critical diseases, specifically when the patient was young or near their age. This theme can be broken down into subcategories: disease's characteristics and patient's characteristics.

#### 3.4.1. Disease's Characteristics

Most participants tended to reflect on situations in which they faced rare, emergency, new, and incurable diseases. They said that, in facing such diseases, they encountered many challenges that led them toward reflecting on their experience.

In this relation, one of the participants (P2) stated that

when I encounter a new and rare case my mind gets busy about what the disease is and many other questions. It is my duty to patient education. Therefore, I assess my experience about the disease.

#### 3.4.2. Patient's Characteristics

According to participants' experiences, patients' characteristics such as their age and their level of awareness of their disease provided more opportunities for better reflection.

One of the participants (P19) argued that

some of my patients have a lot of information about their disease and ask more specific questions about their caring affairs, which caused me to try more to be a step ahead in dealing with such diseases or at least not to know less than my patients.

Another participant (p4) argued that

my patient had a lot of information regarding his disease. There always remains this concern that I may encounter a patient who knows more than I do about his disease, and I may not be able to respond to his questions, and this does not allow me to be indifferent after training.

Another participant (P10) pointed to the age of his patient and said that

my patient had a Guillain-Barre and was 19 years old. It was a rare disease that may happen to me. This caused me to reflect more on it and be encouraged to search about its cure. Such conditions get me involved, and I follow them after training to acquire information about it.

### 3.5. Competent Trainer

In the present study, from participants' point of view, trainer-related factors have essential roles in facilitating students' reflection. They believe that using proper training and supervising method by the trainer in the clinical setting leads them to get more involved in their experience and do activities related to their experience. This theme can be broken down into subcategories: effective training and effective supervision.

#### 3.5.1. Effective Training Methods

According to the participants, using training methods such as case method, questioning techniques, expressing experiences, clinical scenarios, and group discussion leads most of them to reflect. From participants' point of view, using the above method stimulates dynamic thinking and paves the way for reflecting on clinical experiences.

In relation to case method, one of the participants (p3) stated that

some trainers divide the nursing activities among different nursing students, for example; one nurse is responsible for giving the drug; the other one is responsible for checking the signs so that we do not get involved in the disease and its diagnosis. Nevertheless, when we are given a case, and they use case method, we get involved in the patient's problem to see what we know and what we do not know about it and what we need to complete because we are responsible for the disease.

In relation to questioning technique, another participant (P7) said that

whenever I reported about my patient, my trainer would ask me a lot of questions that I would realize that I need to know a lot more for a good health care. And I focused on that case until the next week.

#### 3.5.2. Effective Supervision

Participants emphasized that trainers would encourage reflection in them by supervising their performance in the clinical setting and providing duties related to the training and effective feedback. In this regard, one of the participants (P9) said that

I am as a raw dough in such situations. I want to be shaped in my trainer's hand. And it is my trainer who has to shape me. He is alleged to teach me fishing. By the assignments that he sets for me and his supervising, he can define my path and create dynamic thinking in me as a student. It shouldn't be in a way that I go there and gain everything just there, or I go there and gain everything incompletely and have no motivation to go back toward my experiences and complete them.

### 3.6. Effective Human Relations

Most students emphasized the central role of interpersonal relationship and constructive interaction with other health care team personnel in creating a working environment that supports reflection. They believed that communication factors lead to the increased need for thinking about clinical experiences and improving the reflection process. This theme can be broken down into subcategories: interpersonal relations and constructive cooperation.

#### 3.6.1. Interpersonal Relations

According to participants, respectful and friendly relationship between personnel has a significant impact on reflecting on their performance.

One of the participants (P17) stated that

in female ward at the hospital, the personnel had a good relationship with us, and we were comfortable with them, and they would guide us about diseases and encouraged us to study more about them. I have Pease when I reflect on my experience of female ward.

Another participant (P13) said that “one of the personnel would come with us and check our work, and in case we had a lot of problems, he would behave in a way that the patient would not notice it. I come back to my experience under the influence good behavior of personnel.”

#### 3.6.2. Constructive Cooperation

According to the participants, cooperation and interaction with other disciplines in clinical setting and sharing knowledge and experience in relation to dealing with patient's problem help the participants to follow their clinical experience and reflect on it. One of the participants (P14) said that

whenever the physician wanted to check the patient's reflexes, he would show us the reflexes and deformities and also the experiments. Thus, when a physician cares about the nurses, and wants to nurse students to understand the cases, it stimulates me and increases my interest and attempts, and it encourages me not to be indifferent in relation to different cases.

Another participant (P16) stated that

when we visit patients with other students in different fields of study and hear their speech in the case, we remember that we have already studied them but have forgotten them or remember just some of them, we try to attend with more preparation and study about them.

Another participant (P13) stated that

it is important who is beside us. Fortunately, I am in a group whose members are so curious and tend to study and reflect. Therefore, being in such a group stimulates me.

## 4. Discussion

The present paper reports the findings from the study in which nursing students in Iran were interviewed and in-depth and qualitative methods were used. Data analysis indicated that facilitating reflection factors have a wide variety in different personal and educational levels. In personal level, motivational factors have an essential role in facilitating students' reflection on clinical experiences. Following the need to gain practical knowledge, accountability to patients, and students' tendency affected by their beliefs facilitates reflection on their clinical experiences and their growth and development. Ayduk and Kross have also looked at the tendency for individuals to spontaneously take a self-distanced or immersed perspective when they think about a past experience [19]. Motivation and tendency are considered as factors directing human activities. Motivation is an internal force that consciously or unconsciously leads the individual toward satisfying his physiological and psychological needs toward a specific behavior [20]. In nursing discipline, which deals with human life, being reduced of motivation and tendency not only leads to problems to the nurse but also has destructive effects on the health of society and results in a waste of time and money [21]. The results of study done by Najafi et al. indicated that communicative problem with nursing students is one of the most important factors in declining their motivation and interest toward nursing. They indicated that a good nursing practice after reflection is related to being motivated [22]. Similarly, another researcher stated that positive feelings, feelings of being valuable and accepted, play important roles in increasing self-confidence and motivation. In this study, the quality of trainer's educational relationship with students to cause motivation is emphasized [23]. Chabeli and Muller (2004) in a qualitative study stated that emotional skills, including acceptance, responsiveness, valuation, organization, and internalization, are known as the prerequisites of reflective thinking in clinical nursing education [24]. Johns (2004) has mentioned some mental characteristics as the prerequisites of reflection; these factors include curiosity, commitment, and intelligence, contrary to negative properties of mind such as defensive mind, habits, strength, and ignorance [25]. Accordingly, students' tendency and motivation are essential in using reflection skills and reflection does not occur without students' will and tendency. The participants of this study also emphasized the role of their tendency in reflecting on clinical experiences.

Data analysis indicated that competent trainer could facilitate students' reflection process in clinical education. By expressing their memories and experiences, using Socratic dialogues and guiding group discussions, trainers could facilitate students' reflection on their experiences. Henderson et al. (2002) indicated that promoting event analysis should be done along with other factors to facilitate reflection in the medical area, ensuring that trainers are trained adequately and provide a reliable environment for students and have effective relationship with their students [13]. According to Seibert and Daudelin (1999), effective reflection is impossible without providing conditions such as independence in practice, proper supportive structure, effective feedback, importance of activity, and appropriate challenging cases. Providing such conditions is the responsibility of the trainer [26]. Another study has shown that students need the trainer help to identify issues that need reflection. They also need the guidance, direction, and supervising of their trainer during the process of reflection [27]. Dr. Cox considers the trainer role as an important factor after students' clinical exposure and believes that the trainer should listen to the students' speech, criticize their reasoning and thoughts, evaluate their performance in patient exposure from different aspects, and give immediate and appropriate feedback. This evaluation includes time spent, expressions used, reaction to patient's speech, consideration of logical course of conversation, and attention to technical skills. Trainer as an educational designer should encourage students to learn more from other resources and similar patients [28]. Encouraging students to talk about their findings and also commenting on the reasons make students talk about their shortcomings, weaknesses, and ambiguity and confusion courageously and not hide their potential negligence.

In this study, students argued that new experiences during the training course provide the context for reflection. Many researchers believe that the development of reflection and cognitive and metacognitive skills occurs when a proper level of complex and challenging situations is presented as part of students' learning experience [26, 29]. Rogers (2001) pointed to stimulating, unusual, and perplexing events as one of the most important components of reflection practice [30]. Encountering challenging situations leads to a lack of interaction and the emergence of a feeling of need. Other researchers also pointed to the fact that reflection occurs as a result of being aware of a need and usual need cluttering. This issue occurs in response to complex and unusual situations that cannot be solved easily [7, 31, 32]. Dewey's definition of reflection as exploring solutions for problems is consistent with the above facts [33]. Therefore, although today nursing students face different complex situations in caring patients, there is a broader context for developing reflection.

Effective relations were another facilitating reflection stated by students. According to views of the participants of the study, providing an appropriate environment and encouraging students to present their ideas and interacting with them to challenge them through discussions about experienced clinical situations facilitate reflection. Effective relationship and constructive feedback between the trainers, personnel, experts, and students are the backbone of learning in clinical setting [34]. Open and friendly relationship helps individual identification [35], so that students can reflect better on their experiences and share them [13]. Mongwe (2009) also noted that students argued that the existence of desirable relationship among trainers, personnel, students, and patients results in a pleasant learning environment and acquiring appropriate experiences in clinical practice [36]. Furthermore, in de Guzman et al. study, students referred to interpersonal relationship as the most important characteristic of an efficient trainer [37]. Therefore, creating the conditions for facilitating communication in clinical setting provides facilitating reflection conditions on students' clinical experiences.

## 5. Conclusions

The results of the study indicate that nursing students experienced reflection as a result of motivational factors and educational factors. According to the findings, it is recommended that the trainers prepare students for reflection on practical experiences with a specific focus on their relations and motivational factors. The cooperation among trainers, physicians, nursing personnel, and nursing students can provide a good condition for students' reflection on their experiences through appropriate interaction and provide a desirable supportive atmosphere. Conducting other researches regarding facilitating reflection in nursing education from the point of view of trainers, physicians, and nursing personnel is also recommended.

## 6. Limitation and Future Research

This study had some limitations that should be considered. This study was conducted among nursing students of just two universities, so its findings are not intended to be generalized to other students or universities. Another limitation in the present study was limited to experience of nursing students; thus, future studies should consider looking at other subthemes with examining nursing educators' experiences of facilitating reflection in clinical setting.

## Figures and Tables

**Table 1:** A list of main themes and subcategories extracted from the data.

Main themes	Subcategories
Motivation to reflect	Desire to know more
	Sense of accountability
	Self-actualizing tendency
	Personal values

Complex experiences	Disease characteristics
	Patient's characteristics

Competent trainer	Effective training methods
	Effective supervision

Effective human relations	Interpersonal relations
	Constructive cooperation
